# Supplementary material for: PCR bias impacts microbiome ecological analyses
Source: PLoS Comput Biol. 2026 Jan 27;22(1):e1013908. doi: 10.1371/journal.pcbi.1013908 (PMC12885373; doi:10.1371/journal.pcbi.1013908)
Supplement: S1 Appendix — This supplement contains theorems establishing perturbation-invariant estimands, which remain robust under PCR-induced bias. It also includes corollaries on differential log-ratio abundances and the Aitchison distance, providing theoretical support for compositional analyses in microbiome studies. (PDF) [file pcbi.1013908.s001.pdf]

Supplementary to PCR Bias Impacts Microbiome Ecological Analyses

Dharmik Rathod[1] and Justin D. Silverman[1,2,3,4,*]

[1]College of Information Sciences and Technology, The Pennsylvania State University
[2]Department of Statistics, The Pennsylvania State University
[3]Department of Medicine, The Pennsylvania State University
[4]Institute for Computational and Data Science, The Pennsylvania State University
[*]Corresponding Author, JustinSilverman@psu.edu

A Appendix A: Supplementary Proofs

Theorem 1 (Perturbation-Invariant Estimands are Invariant to PCR Bias). *Let $\Psi \in \mathbb{R}^{(D-1)\times D}$ be a rank $D-1$ contrast matrix with columns summing to zero, and suppose the observed compositional vector $w_n \in S^D$ for sample n follows the linear log-contrast model from Eq (2):*

$$\Psi \log w_n \;=\; \Psi \log a \;+\; x_n \, \Psi \log b,$$

where $a \in S^D$ is the true (pre-amplification) composition and $b \in \mathbb{R}^D$ is the vector of taxon-specific PCR efficiencies.

Let $\theta(\cdot)$ be any estimand that is perturbation invariant, i.e.,

$$\theta(\pi) \;=\; \theta\big(\phi^{-1}\big(\phi(\pi) \;+\; \gamma \mathbf{1}_N^T\big)\big) \quad \textit{for all } \gamma \in \mathbb{R}^{D-1},$$

where $\pi \in S^D$ is any compositional vector, $\phi(\cdot)$ is a log-ratio transform, and $\phi^{-1}(\cdot)$ its inverse.

Then θ is invariant to PCR bias:

$$\theta(w_n) \;=\; \theta(a).$$

Proof. From the PCR bias model:

$$\Psi \log w_n \;=\; \Psi \log a \;+\; x_n \, \Psi \log b.$$

Define the log-ratio transformed vectors:

$$\phi(w_n) \;=\; \Psi \log w_n, \qquad \phi(a) \;=\; \Psi \log a.$$

Then PCR bias appears as an additive perturbation in log-ratio space:

$$\phi(w_n) \;=\; \phi(a) \;+\; \gamma, \quad \text{with } \gamma \equiv x_n \, \Psi \log b \in \mathbb{R}^{D-1}.$$

By the perturbation-invariance property of θ:

$$\theta(w_n) \;=\; \theta\big(\phi^{-1}(\phi(a) + \gamma)\big) \;=\; \theta(a).$$

Thus, θ is unaffected by PCR bias under this model. □

Corollary 1 (Differential Log-Ratio Abundance is Invariant to PCR Bias)**.** *Let the true differential log-ratio abundance of two taxa between two biological conditions $z_n \in \{0,1\}$ be defined as:*

$$\tau(a) \;=\; \underset{n:z_n=1}{\text{mean}} \log\left(\frac{a_{d_1}}{a_{d_2}}\right) \;-\; \underset{n:z_n=0}{\text{mean}} \log\left(\frac{a_{d_1}}{a_{d_2}}\right),$$

where $a \in S^D$ is the true (pre-amplification) composition. Suppose the observed composition $w_n \in S^D$ follows the PCR bias model from Eq (2):

$$\Psi \log w_n \;=\; \Psi \log a \;+\; x_n \, \Psi \log b,$$

with $b \in \mathbb{R}^D$ the vector of taxon-specific PCR efficiencies and x_n the number of PCR cycles.

Then the differential log-ratio abundance is invariant to PCR bias:

$$\tau(w_n) \;=\; \tau(a).$$

Proof. The estimand τ depends only on differences in log-ratios of two taxa (d_1 and d_2) between groups. Adding a constant perturbation γ to the log-ratio space shifts all coordinates by the same amount, but such a constant cancels when computing log-ratio differences and group means. Formally:

$$\tau\big(\phi^{-1}(\phi(w_n)+\gamma)\big) \;=\; \tau(w_n).$$

Thus, τ satisfies the perturbation-invariance condition of Theorem 1. Applying the theorem:

$$\tau(w_n) \;=\; \tau(a).$$

□

Corollary 2 (Aitchison Distance is Invariant to PCR Bias)**.** *Let the true Aitchison distance between two communities n_1 and n_2 before PCR amplification be:*

$$\delta(a_{n_1}, a_{n_2}) \;=\; \sqrt{\sum_{d=1}^{D-1} (\Psi \log a_{n_1} \;-\; \Psi \log a_{n_2})_d^2},$$

where $a_n \in S^D$ is the true (pre-amplification) composition and Ψ is a rank-$(D-1)$ contrast matrix with columns summing to zero. Suppose the observed composition $w_n \in S^D$ follows the PCR bias model from Eq (2):

$$\Psi \log w_n \;=\; \Psi \log a_n \;+\; x_n \, \Psi \log b,$$

with $b \in \mathbb{R}^D$ the vector of taxon-specific PCR efficiencies and x_n the number of PCR cycles.

Then the Aitchison distance is invariant to PCR bias:

$$\delta(w_{n_1}, w_{n_2}) \;=\; \delta(a_{n_1}, a_{n_2}).$$

Proof. The Aitchison distance depends only on pairwise differences in log-ratio coordinates:

$$\delta(w_{n_1}, w_{n_2}) \;=\; \sqrt{\sum_{d=1}^{D-1} (\phi(w_{n_1})_d \;-\; \phi(w_{n_2})_d)^2}, \quad \text{with } \phi(w_n) = \Psi \log w_n.$$

Adding a constant perturbation γ shifts all log-ratio coordinates by the same amount, but such a constant cancels when taking differences:

$$\phi(w_{n_1}) + \gamma \;-\; \phi(w_{n_2}) - \gamma \;=\; \phi(w_{n_1}) - \phi(w_{n_2}).$$

Thus:

$$\delta\big(\phi^{-1}(\phi(w) + \gamma)\big) \;=\; \delta(w).$$

The Aitchison distance therefore satisfies the perturbation-invariance condition of Theorem 1. Applying the theorem:

$$\delta(w_{n_1}, w_{n_2}) \;=\; \delta(a_{n_1}, a_{n_2}).$$

□
