# Supplementary figures and images for: PCR bias impacts microbiome ecological analyses
Source: PLoS Comput Biol. 2026 Jan 27;22(1):e1013908. doi: 10.1371/journal.pcbi.1013908 (PMC12885373; doi:10.1371/journal.pcbi.1013908)

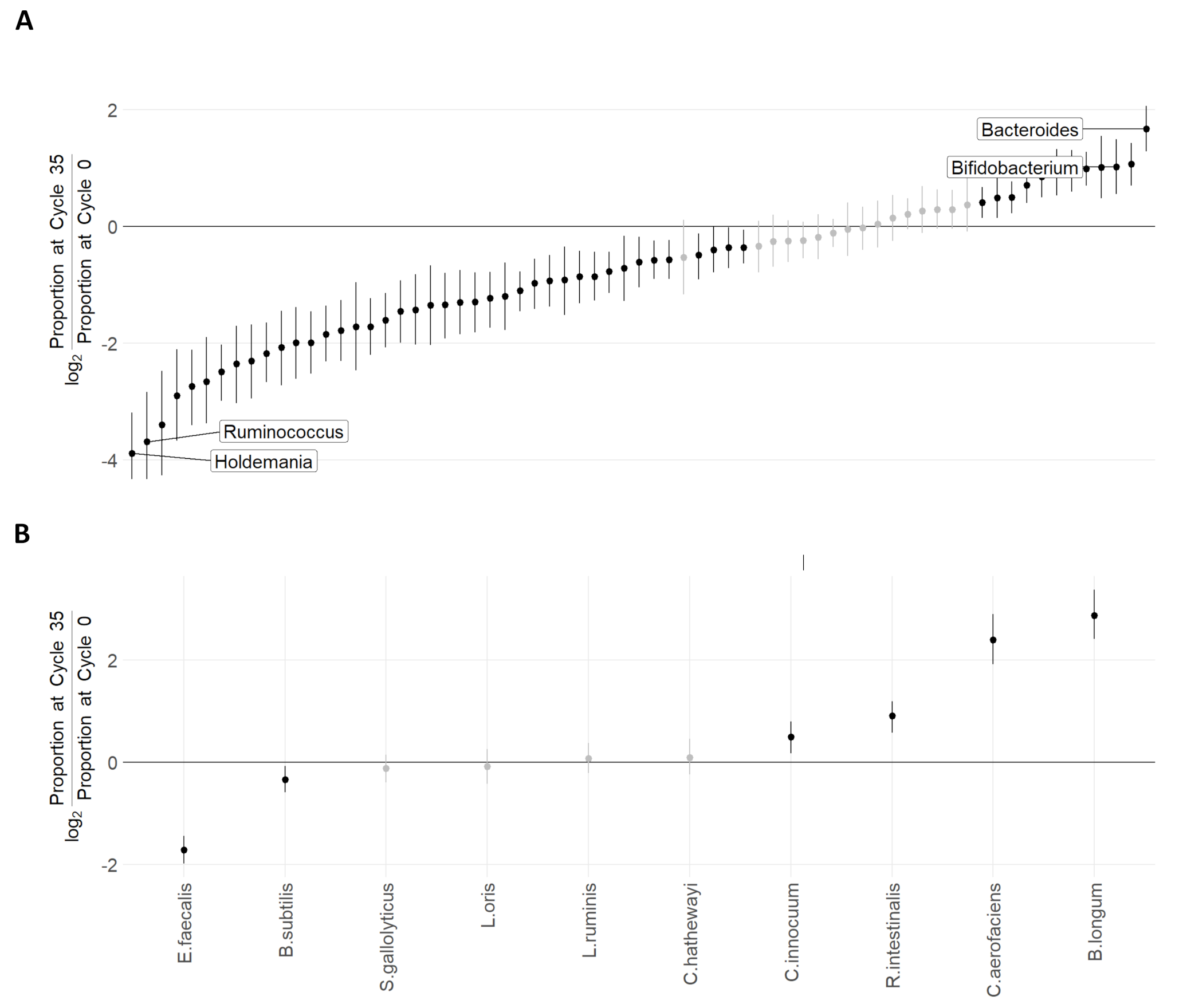

Supplement: S1 Fig — Bias was quantified as the log2-ratio of each taxon’s estimated relative abundance after 35 PCR cycles (cycle 35) to its estimated pre-amplification abundance (cycle 0) for (A) human gut microbiota and (B) mock communities from Silverman et al. [8]. A log2 value of 2 indicates a fourfold overestimation due to PCR, whereas -2 indicates a fourfold underestimation. Points show posterior medians, and bars denote 95% credible intervals. Taxa with credible intervals excluding zero (statistically credible bias) are highlighted in black. (TIFF) [file pcbi.1013908.s002.tif]

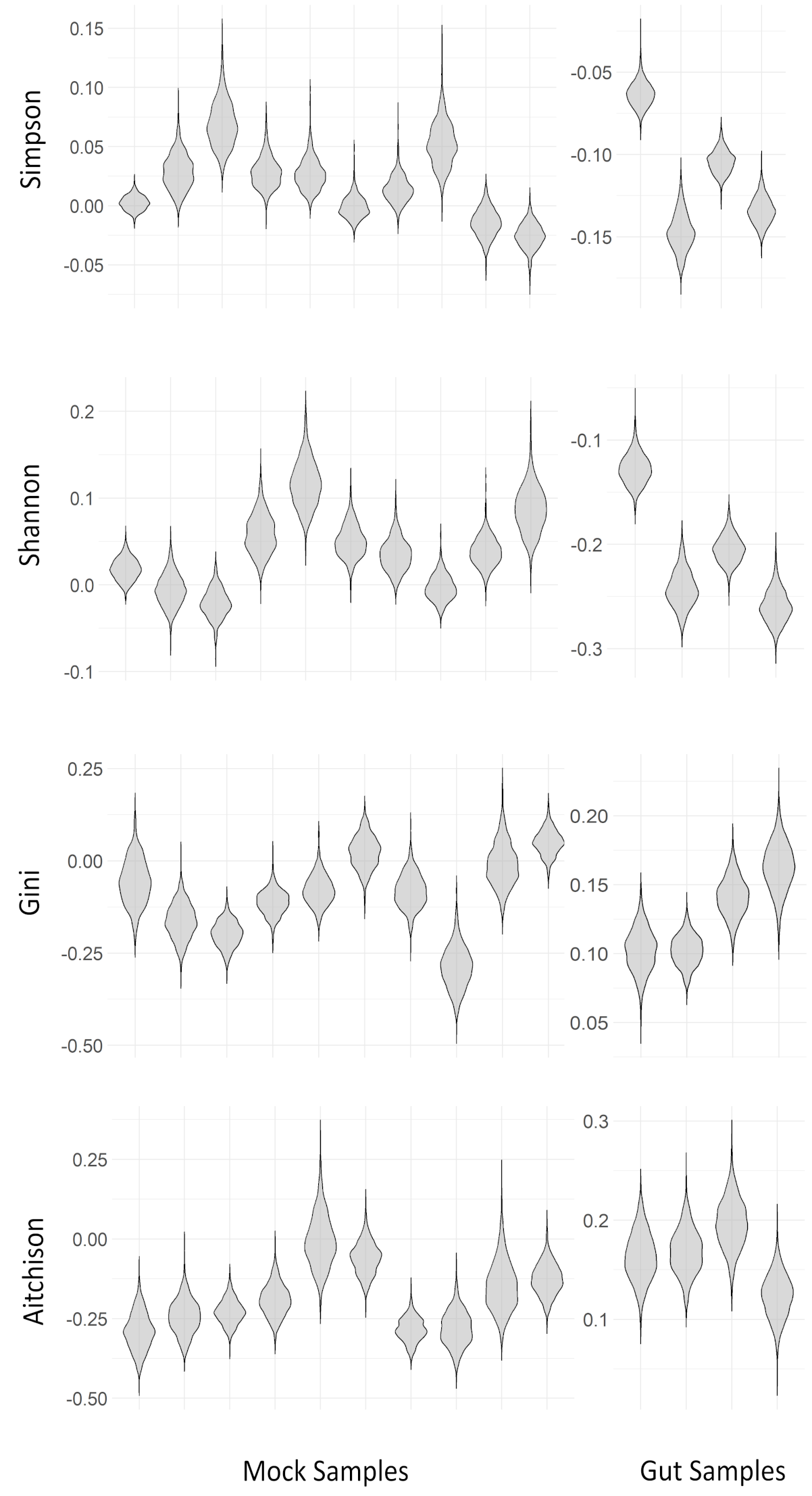

Supplement: S2 Fig — Violin plots show posterior distributions of sample-specific relative bias for four α-diversity metrics (Simpson, Shannon, Gini, Aitchison) across gut and mock communities. The shapes of the distributions highlight that the magnitude and direction of PCR bias vary systematically across metrics and differ between controlled mock communities and complex gut communities. Together, these patterns demonstrate that PCR amplification can introduce non-uniform distortions that depend both on the diversity metric and on underlying community structure. (TIFF) [file pcbi.1013908.s003.tif]

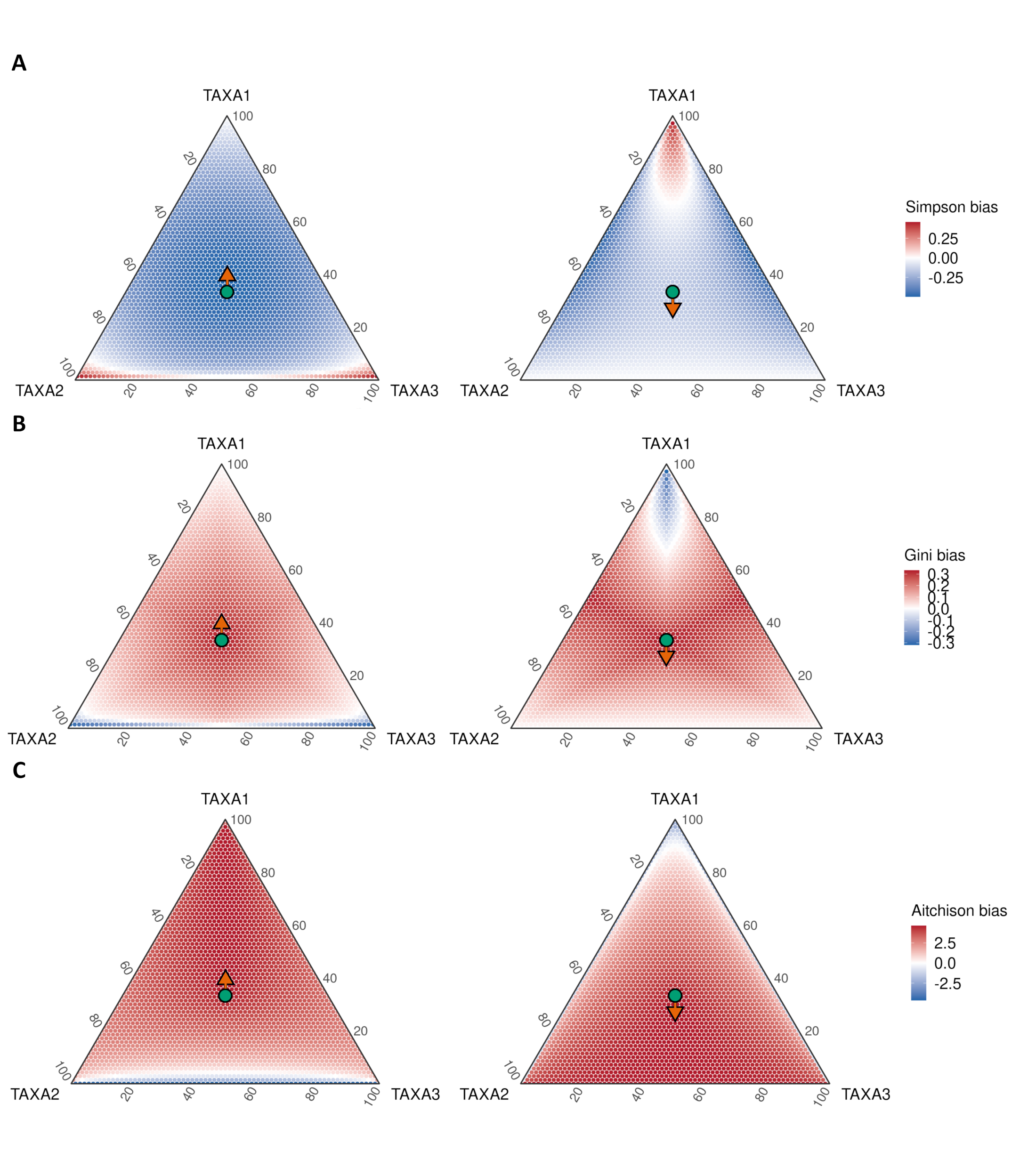

Supplement: S3 Fig — Ternary diagrams show PCR-induced changes in (A) Simpson diversity, (B) Gini coefficient, and (C) Aitchison norm after 35 cycles under two different bias vectors (orange arrow). On the left the PCR bias preferentially amplifies TAXA 1 relative to the other two taxa, whereas on the right, TAXA 2 and 3 are preferentially amplified over TAXA 1. Each point represents an initial pre-amplification composition in a three-part simplex, colored by the change in the corresponding metric after PCR. The green dot marks the unbiased origin, and the orange arrow indicates the direction and magnitude of the bias vector. Even small differences in amplification efficiency produce highly non-uniform distortions, with bias magnitude varying systematically across the compositional space. (TIFF) [file pcbi.1013908.s004.tif]

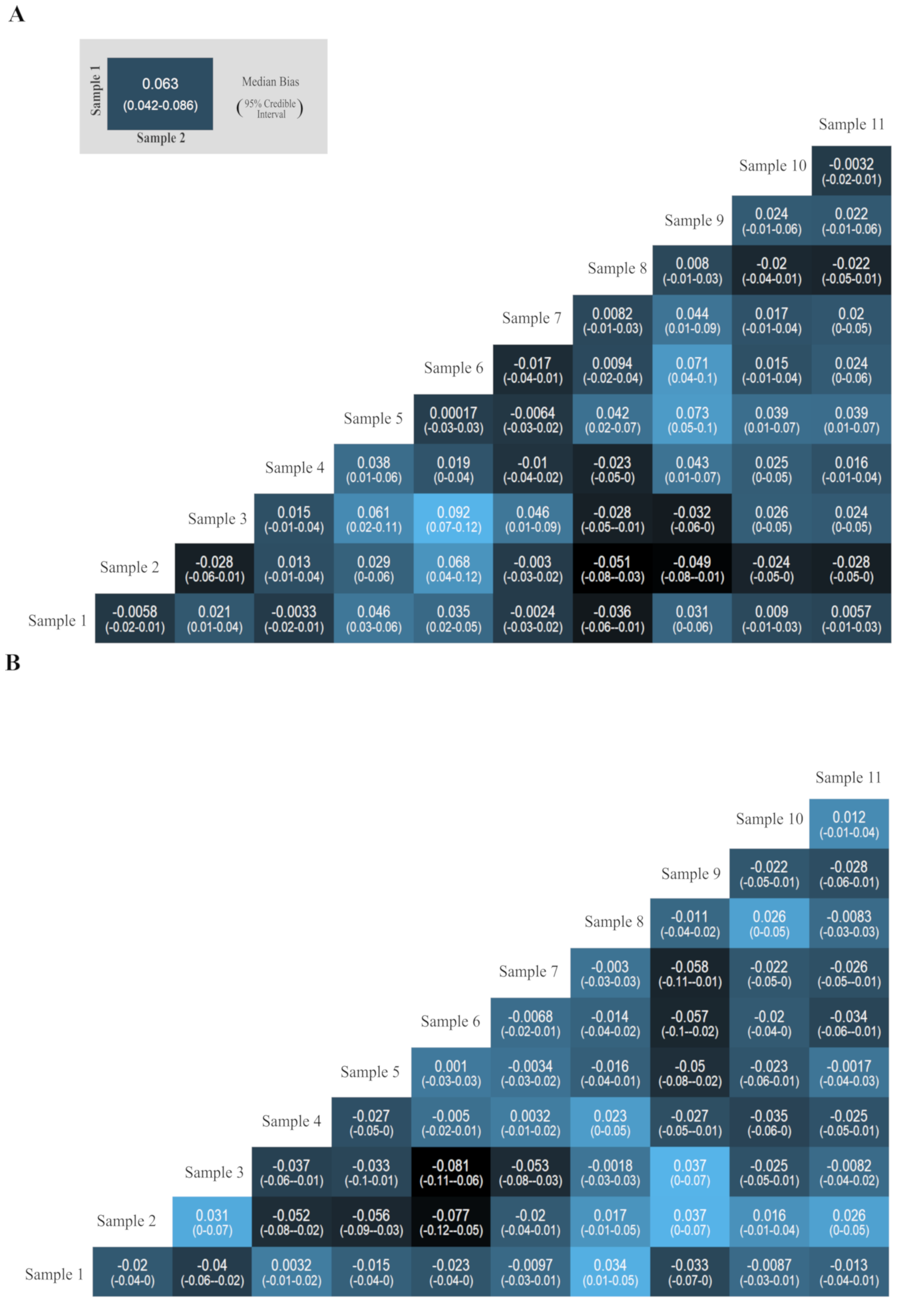

Supplement: S4 Fig — Heatmaps show changes in (A) Weighted UniFrac and (B) Bray-Curtis distances between pairs of mock community samples after 35 PCR cycles relative to their pre-amplification (cycle 0) values. Each tile shows the posterior median change, with 95% credible intervals in parentheses. Rows and columns correspond to sample indices. Variation in bias across pairs indicates that PCR amplification introduces systematic and sample-specific distortions in inter-sample dissimilarities. (TIFF) [file pcbi.1013908.s005.tif]

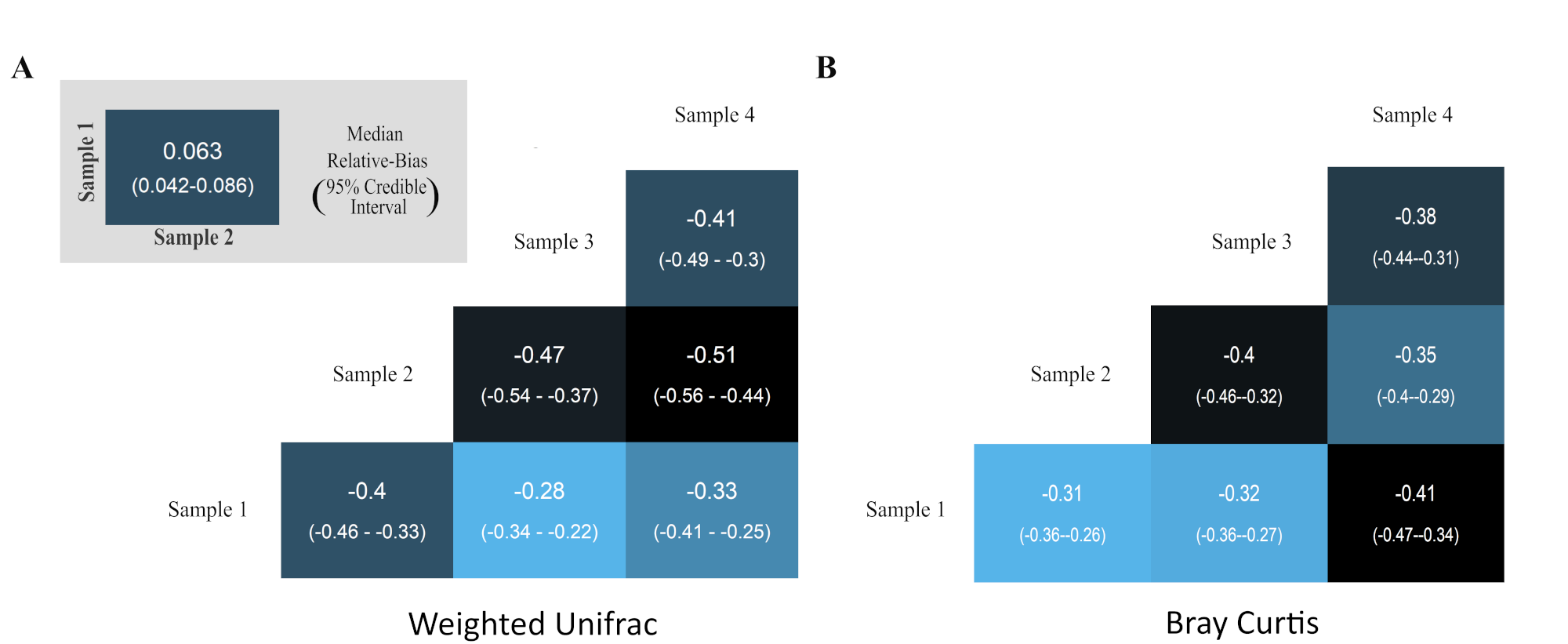

Supplement: S5 Fig — Plots show posterior distributions of pairwise relative bias for Bray-Curtis and weighted UniFrac distances, summarizing how PCR alters dissimilarity between samples. The distributions reveal substantial variation in magnitude of distortion across sample pairs, reflecting the dependence of PCR-induced changes on the underlying community compositions being compared. These patterns indicate that PCR amplification can introduce non-uniform, comparison-specific distortions in β-diversity estimates. (TIFF) [file pcbi.1013908.s006.tif]

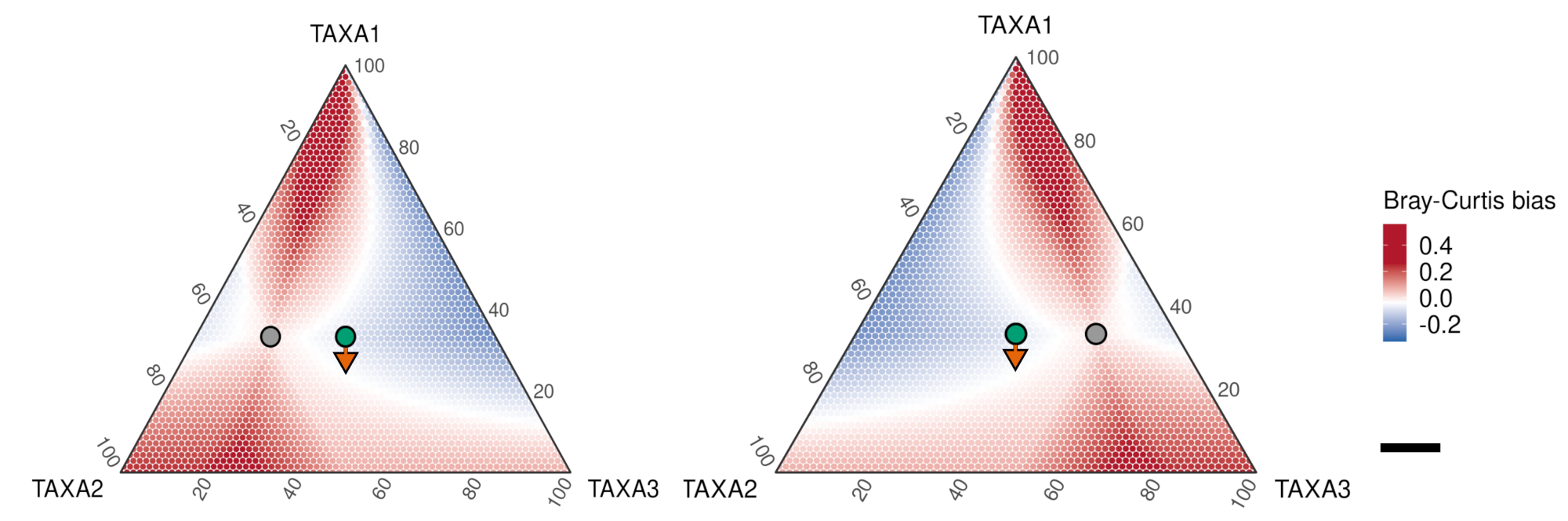

Supplement: S6 Fig — Change in Bray-Curtis distance is shown as the second community varies across the simplex relative to a fixed reference composition, with PCR bias held constant. Colors indicate the difference between the Bray-Curtis value at 35 PCR cycles and its true pre-amplification value. The green dot marks the unbiased origin, the orange arrow indicates the direction of PCR bias, and the grey circle marks the fixed reference community. (TIFF) [file pcbi.1013908.s007.tif]
