# Supplementary material for: PCR bias impacts microbiome ecological analyses
Source: PLoS Comput Biol. 2026 Jan 27;22(1):e1013908. doi: 10.1371/journal.pcbi.1013908 (PMC12885373; doi:10.1371/journal.pcbi.1013908)
Supplement: Attachment — Submitted filename: Response to Reviewers.pdf [file pcbi.1013908.s009.pdf]

Response to Reviewers: PCR Bias Impacts Microbiome Ecological Analyses

December 3, 2025

We thank the reviewers for their helpful comments about our manuscript. The response to each point can be found below. For convenience, we have provided a copy of the manuscript with changes marked. Any additions to the manuscript are marked in blue, and any deletions are marked in red. Below reviewer comments are marked in blue, and our responses in black. All line and page references match the version of the manuscript with marked changes.

Reviewer 1

Summary: Measuring alpha-diversity (within-sample) and beta-diversity (between-samples) are key aspects of analyzing the structure of microbial communities. 16S sequencing is a ubiquitous assay for measuring the composition of these microbial communities, but relies on PCR amplification, which can show differential amplification based on the primers used, resulting in PCR bias. In particular, Non-Primer Mismatch (NPM) persists through all cycles of PCR (while primer-mismatch bias is present only in the initial cycles). Here, Rathod and Silverman use their previously developed model (Silverman et al 2021) to investigate the impact of NPM bias on downstream diversity-based analyses that are frequently used. They prove some fundamental results about which types of measures are susceptible to this bias and demonstrate the possible implications of using these measures.

Overall impression: The paper is generally well-structured and addresses a fundamental question about the tools used to interrogate the composition of microbial communities. I found this paper to be very compelling, but there are opportunities for improving the presentation of the statistical model and the results.

We thank the reviewer for their support and thoughtful comments which have improved the paper.

Major issues:
My biggest complaint is the lack of clarity around the simplex figures. What are the defining parameters for the two different scenarios? In figure 2 is it just the orientation of the PCR bias represented by the orange arrows? What is the orange dot? What does it mean for green arrow to “mark the unbiased origin”? Each metric shows bias around the green dot. Does the green dot not represent where each of the taxa are present at a 1/3 proportion? It would be helpful to be explicit around what the PCR bias parameters are in these simulations and what is different between scenarios.

Thank you for raising these points. We have both simplified these figures to make them more intuitive and added an inline inset figure that visually annotates the simplex diagrams. Below we

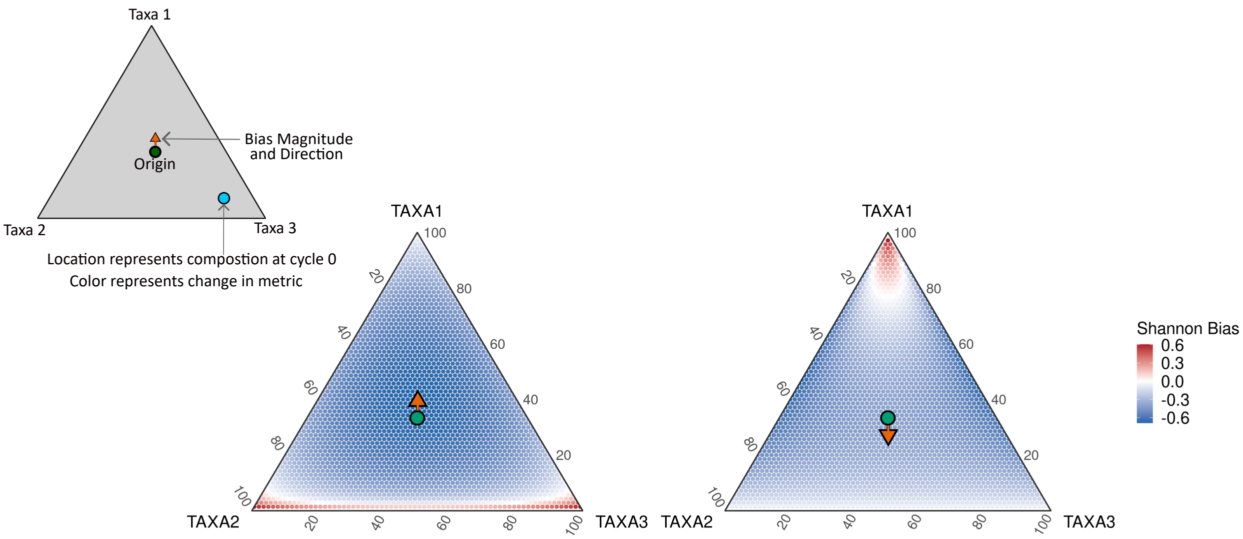

Figure 1: Bias in Shannon index varies across the compositional space. Simplex plots illustrate how PCR amplification alters Shannon diversity under two different bias scenarios. On the left the PCR bias preferentially amplifies TAXA 1 relative to the other 2 taxa , whereas on the right, TAXA 2 and 3 are preferentially amplified over TAXA 1. Each point represents an initial (pre-amplification) three-taxon composition, and its color indicates the change in Shannon diversity after 35 PCR cycles. The orange arrow shows the direction and magnitude of the PCR bias vector in composition space, and the green dot marks the unbiased origin. Even small differences in amplification efficiency produce highly non-uniform distortions, with the magnitude of bias depending strongly on the initial community composition.

show the new version of Figure 2 as an example, our modifications for Figure 4 are analogous.

I find the data used in this paper to be quite useful and didactic. I am not knowledgeable about current work being done in metagenomics, but is there a scenario where incorrect conclusions have been drawn due to the presence of high PCR bias? Finding such an example and demonstrating that a perturbation invariant measure (or otherwise correcting the analysis) would draw the correct conclusions could be quite impactful. This is a question that could potentially improve the manuscript and not a requirement for publication.

We have added a discussion paragraph outlining how our approach may be extended to metagenomic studies and other applications that rely on PCR-based measurement technologies.

> Although our analyses focus on 16S rRNA gene sequencing, the framework developed here likely extends to other sequencing applications in which PCR amplification is used during library preparation. Many shotgun metagenomic and viromic workflows employ PCR–especially for low-input samples–and targeted amplicon, immune repertoire, and RNA-seq protocols rely on amplification steps that can introduce sequence- or taxon-specific distortions in observed abundance profiles. While we are not aware of published studies showing that PCR bias alone has produced incorrect biological conclusions in these contexts, such outcomes are plausible given the ubiquity of PCR and the sensitivity of many diversity metrics to amplification-induced perturbations. Applying perturbation-invariant measures or bias-correction methods in these broader settings may help identify and mitigate such effects. More generally, the distinction between perturbation-sensitive and perturbation-invariant estimands provides a unifying framework for improving robustness in sequencing-based inference and may prove valuable for PCR-dependent analyses across metagenomics, transcriptomics, and immune repertoire

profiling.

Minor issues:

I have a large number of minor issues, but the majority of them are related to writing/figures and can be resolved with little-to-no additional analyses.

The linear log-contrast model notation is a little confusing as written in section 2.1 leading up to equation (2). Rather than introducing everything as vectors and forcing the reader to think about row vs column vectors and then and saying the contrast vector can be viewed as a matrix, simply give the 2x1 matrix formulation from the beginning. Given the multivariate linear model $\alpha = x_n\beta\ \Sigma$, the multinomial GLM is a natural model.

We thank the reviewer for the suggestion. We have made this change and it makes this section much more concise.

In general, I would err on the side of defining every symbol used (ϕ for example, is undefined) and making it clear what the dimensions of every matrix are (for example, when taking the vector outer product to obtain an N x D-1 matrix) rather than letting some dimensions be implicitly denoted.

Thank you for pointing this out. We have revised the manuscript to explicitly define all symbols (including ϕ) at their first appearance and to clearly state the dimensions of all matrices involved in the derivations.

You state that you use the fido R library to estimate both η and Σ . Can you give a short, didactic description of how fido works?

We have added the following description of fido in the Methods section.

> All model fitting was performed in `R` using the `fido` package [Silverman et al., 2022]. *fido* implements scalable Bayesian inference for multinomial logistic-normal models using the collapse–uncollapse sampler introduced in Silverman et al. [2022]. In brief, *fido* exploits a marginal-Laplace inference strategy: (i) it first *collapses* the model by analytically marginalizing over the regression parameters $\boldsymbol{\alpha}$, $\boldsymbol{\beta}$, and the covariance $\boldsymbol{\Sigma}$; (ii) it then approximates the marginal posterior of the latent log-ratio variables using a *Laplace approximation*; and (iii) it *uncollapses* by conditioning on samples from this Laplace approximation to generate posterior samples of $\boldsymbol{\alpha}$, $\boldsymbol{\beta}$, and $\boldsymbol{\Sigma}$. This approach avoids high-dimensional MCMC over latent variables and enables fast and accurate posterior inference [Silverman et al., 2022].

For the general audience of PLoS Computational Biology, would be nice to have an intuitive explanation in the text explaining Corollary 1/the perturbation invariant property. Additionally, the theorems/corollaries are quite natural, but their presentation in the theorem-proof manner might turn away the general reader. It might be helpful to intuitively present these results in plain language.

Thank you for this suggestion. In response, we have added the following intuitive, plain-language explanation of the perturbation-invariant property in the main text.

> Informally, an estimand is perturbation invariant if its value does not change when all samples are subjected to the same compositional perturbation–that is, when their log-ratio representations are shifted by the same vector in log-ratio space.

Figure 1 does not have quantitative labels for the bias posterior distributions. These would be helpful, at least for the x-axes.

Thank you for the suggestion. We have updated Figure 1 to include quantitative x-axis labels for the posterior distributions of the bias parameters.

Could it be useful to define a relative bias, i.e. the current Bias divided by the estimated log-ratio composition before amplification (and the same question for the beta diversity bias)?

Thank you for raising this point. We agree that defining a relative bias, such as the estimated PCR bias divided by the inferred log-ratio composition prior to amplification, could provide additional interpretability in certain contexts. In the Results section, we have added the following concise discussion of relative bias, and we have included a supplementary figure to illustrate it in greater detail.

> All four metrics are clearly impacted by PCR bias, with values after 35 PCR cycles substantially differing from their true pre-amplification (0-cycle) values (Figure 2). To further quantify this effect, we also measured the posterior distributions of *relative bias* for each metric in Figure S2 using the ten mock communities and four human gut microbiota samples from Silverman et al. [2021]. Formally, let $\phi^{-1} : \mathbb{R}^{D-1} \to \mathbb{S}^D$ denote any inverse log-ratio transformation and $f : \mathbb{S}^D \to \mathbb{R}^+$ denote an α-diversity metric on the D-dimensional simplex. We define PCR-induced relative bias as:
>
> $$\text{Relative Bias} = \frac{f\left(\phi^{-1}(\boldsymbol{\alpha} + 35 \cdot \boldsymbol{\beta})\right) - f\left(\phi^{-1}(\boldsymbol{\alpha})\right)}{f\left(\phi^{-1}(\boldsymbol{\alpha})\right)}.$$
>
> Across both figures, the magnitude and direction of the bias varies across communities, reflecting its dependence on the underlying composition. As a result, PCR amplification can distort not only absolute estimates of diversity but also comparisons across experimental conditions–that is, differential α-diversity analyses such as assessments of whether diversity differs between cases and controls (e.g., Van Syoc et al. [2022]).

Lines 175-180 point to Figure 1, but do not discuss the actual data presented.

We thank the reviewer for noting this. While the data underlying Figure 1 were introduced in the preceding paragraph, we agree that the paragraph referencing Figure 1 should also more clearly describe the dataset. We have therefore expanded the text to explicitly summarize the samples used:

> The dataset consists of ten mock communities with controlled proportions of ten bacterial isolates and four human gut microbiota samples from distinct artificial gut systems. All the artificial guts were inoculated with a human fecal sampled and cultured ex vivo in a system designed to replicate host physiology.

Lines 181-189 about differential alpha diversity analyses are very interesting in terms of showing how big of a deviation practitioners might experience. It is not necessary to build on this, but I am interested in whether these most extreme differences are typical of what might be observed in practice with real groupings rather than those artificially constructed to be as large as possible.

We have added the following discussion paragraph to address this comment.

> Because the mock community dataset lacked natural experimental groupings, we constructed artificial groupings using an optimization procedure designed to maximize

changes in statistical signal across PCR cycles. This produces a worst-case scenario: it identifies sample configurations that are maximally aligned with the directions of variation imposed by amplification bias. Real biological groupings are unlikely to align this strongly, but partial alignment could still produce noticeable inflation or attenuation of diversity-based signals. The optimized groupings therefore serve as a useful interpretive benchmark, clarifying the specific geometric circumstances under which PCR bias exerts its strongest effects and providing context for evaluating the magnitude of bias observed in empirical studies. Further research using empirical datasets is needed to determine how representative these cases are and to better understand the extent to which PCR bias alters diversity-based conclusions in practice.

Figure 2 and Figure S2 are very helpful didactic figures (maybe in giving an intuitive explanation of what the perturbation invariance is, you could characterize it in terms of this 3-simplex?), but it is not clear what the two different scenarios consist of in terms of PCR bias (i.e. what is the difference in simulation parameters between left and right?). Perhaps I missed the explanation.

In response to this comment, we have added the following to the Discussion to explain perturbation invariance in terms of our simplex plots:

> Intuitively, on the simplex (as in Figure 2), perturbation invariance means that every initial composition experiences zero bias; e.g., Figures 2 or 4 would appear completely white. Such estimands therefore remain stable regardless of the community composition, in stark contrast to standard α- and β-diversity measures whose sensitivity to PCR bias varies across the simplex.

We have updated the figure description to clarify the differences in simulation parameters between the two scenarios as well:

> On the left the PCR bias preferentially amplifies TAXA 1 relative to the other 2 taxa, whereas on the right, TAXA 2 and 3 are preferentially amplified over TAXA 1.

As a final comment on the alpha diversity analysis: since all of the alpha diversity metrics presented are perturbation sensitive, does there exist an alpha diversity metric that satisfies realistic desiderata and is perturbation invariant? Not necessary for publication, but this is a natural question. Further, can any of the commonly used perturbation sensitive metrics be "corrected"?

We have added the following statement to the Discussion to address the reviewer's comment.

> At present, we are not aware of any commonly used α-diversity metric that is perturbation invariant, and whether such a metric can be developed remains an open question.

Similar to my comments around Figure 1, lines 214-218 only minimally discuss the data presented in Figure 3.

Thank you for pointing this out. We have expanded our description of the data shown in Figure 3, and the revised wording is provided below:

> Using the Bayesian multinomial logistic model from Silverman et al. [2021] we analyzed four artificial gut systems, each inoculated with a distinct human fecal sample and cultured ex vivo under conditions designed to mimic host physiology

I think the beta diversity metrics could also have a relative bias metric to inform the interpretation of how large these biases are.

Thank you for this thoughtful suggestion. We agree that defining a relative bias for beta-diversity metrics could help contextualize the magnitude of perturbation-induced distortions. In the revised manuscript, we have added a brief discussion in the Results section describing how such a relative measure could be constructed, along with a supplementary figure illustrating this idea.

> PCR introduced substantial, sample-specific shifts in both metrics, with the magnitude of bias varying considerably across sample pairs. To further quantify these distortions, we examined the posterior distributions of the relative change in each pairwise distance of the same four artifical gut systems, revealing that PCR can either inflate or deflate β-diversity depending on the underlying community compositions being compared (Figure S5). Formally, for a β-diversity metric $g : \mathbb{S}^D \times \mathbb{S}^D \to \mathbb{R}^+$, we define relative bias as:
>
> $$\text{Relative Bias} = \frac{g\big(\phi^{-1}(\boldsymbol{\alpha}_1 + 35\,\boldsymbol{\beta}), \phi^{-1}(\boldsymbol{\alpha}_2 + 35\,\boldsymbol{\beta})\big) \;-\; g\big(\phi^{-1}(\boldsymbol{\alpha}_1), \phi^{-1}(\boldsymbol{\alpha}_2)\big)}{g(\phi^{-1}(\boldsymbol{\alpha}_1), \phi^{-1}(\boldsymbol{\alpha}_2))}$$

In the discussion, the main guidance for practitioners is to use the Aitchison distance and to be wary when using other metrics. Are there other takeaways? Should the field develop new, invariant metrics? Should we move towards sampling with multiple values of the number of cycles of PCR? Can we correct this?

Thank you for raising these important points. We have refined the Discussion to more precisely articulate the practical implications, including considerations about invariant metrics, cycle-number sensitivity, and potential correction strategies. The revised paragraph is provided below:

> Given these findings, we recommend using compositional metrics based on log-ratio transformations—such as the Aitchison distance—whenever possible for analyses of PCR-amplified microbiome data. These methods are perturbation invariant and therefore unaffected by PCR bias. However, the choice of metric should ultimately be guided by the underlying biological question. If the question depends on a perturbation-sensitive metric, then efforts should be taken to quantify and mitigate PCR bias to ensure valid inference. In particular, we recommend performing calibration experiments (e.g., sequencing the same community with different numbers of PCR cycles) to estimate community-specific amplification bias and adjust diversity measures following the approaches described here and in Silverman et al. [2021]. When such mitigation is not possible, results from perturbation-sensitive diversity analyses should be interpreted with caution, both in their absolute values and in between-sample comparisons.

Since PCR is so ubiquitously used, are there other application areas where this work can be extended outside of 16S sequencing?

We believe we have addressed this comment as part of our response to the reviewers second major comment.

Other remarks:

The GitHub repo should have a license and the readme should have some instructions for running the R scripts

Thank you for pointing this out. The GitHub repository now includes a license file, and the README has been revised to provide detailed instructions for running the associated R scripts.

2. Since the log-linear model accounts for “95% of the variation due to PCR bias in both mock

and real 16S rRNA libraries", what accounts for the remaining 5%? Is this 5% safely ignorable, or is it impactful?

We appreciate the reviewer's question. The remaining 5% of unexplained variation reflects a combination of natural measurement noise and small deviations from the idealized log-linear model. Noisy count data, stochasticity in PCR amplification, and sequencing variability all contribute variance that no deterministic model could fully capture. It is also possible that minor nonlinearities exist in the true relationship between cycle number and composition, though our analyses suggest these effects are modest. Given that the model explains approximately 95% of the variance—substantially higher than typical for microbiome models—we believe the residual 5% primarily reflects technical noise rather than systematic, biologically meaningful bias.

You state on the main text that Holdemania is underrepresented by a factor of about 32 in Fig. S1, but in Fig. S1, it's log-ratio is approximately -4 and $2^4 = 16$,so there is approximately 16x at cycle 35 as at cycle 0. Am I making a simple/obvious mistake in interpreting S1 or should 32 be replaced with 16? For Bacteroides, you state a factor of 4 in the main text and the log ratio is 2, and $2^2 = 4$, so these values agree.

Thank you for pointing out this oversight. The correct fold-change for Holdemania is indeed 16 rather than 32, as indicated by the log-ratio in Figure S1. We have updated the main text accordingly.

> Briefly, some taxa, such as Holdemania, are consistently underrepresented by a factor of approximately 16, whereas others, such as Bacteroides, are overrepresented by a factor of about 4.

I noticed that you implemented the alpha diversity metrics yourself. Is there no R package that implements these?

Several R packages do implement common α-diversity metrics. However, we elected to implement these metrics directly to ensure that the calculations matched the precise definitions used in our theoretical framework and to maintain full control over numerical details, especially when integrating with posterior samples from our model. In previous work, we have occasionally found that published software routines differ subtly from the mathematical definitions they reference. For this reason, we implement core estimands ourselves when exact alignment between the theory and computation is essential.

This information is in the caption, but Figure 3 panels A and B could use titles to show which metrics they correspond to.

Thank you for pointing this out. We have modified Figure 3 by adding explicit panel titles for A and B to indicate the corresponding metrics.

Reviewer 2

This is an excellent, well-executed, and highly significant manuscript that makes a substantial contribution to the field of microbiome bioinformatics. The research is rigorous, the concept of "perturbation invariance" is a powerful and elegant theoretical framework that effectively explains and predicts the effects of PCR bias. The study successfully bridges a critical knowledge gap by moving beyond the established impact on relative abundances to provide a systematic evaluation of ecological diversity metrics. The conclusions are well-supported by both theoretical proofs and empirical data, and the practical recommendations are clear and actionable.

We thank the reviewer for their support and thoughtful comments which have improved this manuscript.

The use of an optimization procedure to create groupings that maximize delta R2 is clever for demonstrating potential impact. However, it risks being perceived as a strawman argument, as reviewers may question how often such a worst-case scenario occurs in real studies. Please clearly state that this is a "proof-of-concept" or "worst-case" analysis designed to demonstrate the maximum plausible effect of PCR bias, not necessarily a common one. Discuss factors that would make such a severe distortion more or less likely in real data (e.g., how aligned the true biological signal is with the PCR bias vector). This preempts a potential criticism.

We have added a paragraph into the Discussion clarifying these points.

> Because the mock community dataset lacked natural experimental groupings, we constructed artificial groupings using an optimization procedure designed to maximize changes in statistical signal across PCR cycles. This produces a worst-case scenario: it identifies sample configurations that are maximally aligned with the directions of variation imposed by amplification bias. Real biological groupings are unlikely to align this strongly, but partial alignment could still produce noticeable inflation or attenuation of diversity-based signals. The optimized groupings therefore serve as a useful interpretive benchmark, clarifying the specific geometric circumstances under which PCR bias exerts its strongest effects and providing context for evaluating the magnitude of bias observed in empirical studies. Further research using empirical datasets is needed to determine how representative these cases are and to better understand the extent to which PCR bias alters diversity-based conclusions in practice.

Figures 2 & 4 (Simplex Plots): These are excellent for intuition but currently feel somewhat disconnected from the real data analysis. The manuscript would be stronger if you could directly link the patterns in these theoretical simplices to specific sample pairs from your mock/gut data. For example, could you point to a pair in Figure S3 and explain its high/low bias using the principles illustrated in Figure 4?

Thank you for raising this point. We have expanded the Results section for both alpha and beta diversity to more explicitly link the simplex patterns in Figures 2 and 4 with sample pairs from the mock and gut data. The revised text is included below.

For α-diversity (Figure 2):

> These visualizations help explain why some communities in the mock and gut datasets showed large shifts in differential α-diversity analyses, whereas others were less affected (Figure 1). The mock communities were constructed in a way that naturally produces one or two dominant taxa [Silverman et al., 2021]. As a result, these samples tend to occupy the low-bias, compositionally uneven regions of the simplex—where bias typically leads, for example, to overestimation of Shannon diversity but only by a modest amount. In contrast, several gut samples contain moderately abundant genera that Silverman et al. [2021] identified as strongly over- or under-amplified (e.g., Bacteroides, Blautia, Faecalibacterium). Because these efficiency-sensitive taxa are neither rare nor overwhelmingly dominant, gut communities often lie in the high-bias, higher-richness/higher-evenness regions of the simplex and therefore exhibit substantially larger α-diversity distortions after PCR.

For $\beta-$diversity:

Finally, to build intuition for why some community pairs are more affected than others, we visualized PCR-induced bias using a simplified three-taxon system (Figure 4 and Supplementary Figure S6). This hypothetical example, included for visualization only, illustrates how the magnitude of bias depends on where the two communities lie in the compositional simplex. Bias is smallest when the PCR-induced shift is orthogonal to the axis that characterizes the difference between the two communities. In contrast, Bias is largest when the shift impacts the relative abundance of the very taxa that distinguish the two communities. This helps explain the patterns in Figure 3 and Figure S4. Gut samples can be distinguished by several moderately abundant taxa that Silverman et al. [2021] identified as strongly over- or under-amplified (e.g., Bacteroides, Blautia, Faecalibacterium), placing these communities in high-bias regions of the simplex. In contrast, the mock communities are compositionally uneven and dominated by a small number of taxa whose amplification efficiencies are relatively similar; as a result, they tend to fall in stable, low-bias regions across scenarios. This visualization therefore provides intuition for why PCR-induced bias varies so markedly between the mock and gut datasets.

Figure 1: The violin plots effectively show that bias exists and is sample-specific. However, the "two representative samples" for the bias density plots are not identified in the main text. Why were these chosen? Label them (e.g., "Mock Community A," "Gut Sample B") and briefly justify their selection (e.g., "one showing large positive bias, one showing large negative bias").

Thank you—we have revised the Figure 1 description to justify the choice of the two representative samples; the new text appears below.

Violin plots show posterior distributions of four α-diversity metrics (Shannon, Simpson, Gini, and Aitchison) for *in vitro* and *ex vivo* data, estimated before amplification (0th cycle) and after 35 PCR cycles using the model of Silverman et al. [2021]. Density plots to the right show the posterior distributions of the PCR-induced bias (35-cycle value minus 0-cycle value) for two representative samples chosen because they exhibit the largest difference in PCR-induced bias among all samples.

You correctly state that differential log-ratios (a perturbation-invariant estimand) are unaffected by PCR bias. However, many popular differential abundance tools (e.g., DESeq2, edgeR, ANCOM-BC) do not explicitly use this framework and are applied to raw counts. There is an ongoing debate in the field about the best methods. You may briefly discuss the implication of your finding for the choice of differential abundance methods. Does your work implicitly support the use of log-ratio-based methods (like those built on fido) over others when PCR bias is a concern? A sentence or two would add significant translational impact.

We agree with the reviewer. We have added the following paragraph to the Discussion section:

These results also have implications for differential abundance analyses. Many widely used DA tools (e.g., DESeq2 and edgeR) operate directly on count data or normalized counts, making them perturbation sensitive. In contrast, methods based on log-ratio coordinates–including those implemented in fido–target perturbation-invariant estimands and are therefore unaffected by PCR-induced shifts in relative abundance. Our findings do not prescribe a single "best" method, but they do suggest that log-ratio–based approaches provide greater robustness when PCR bias is present or cannot be experimentally calibrated.

Please formalize the definition of “Perturbation-Invariant Estimand”. Before giving the formal definition, state in plain language: ”An estimand is perturbation-invariant if its value does not change when every log-ratio in the composition is shifted by the same constant value.” Then, explicitly state that PCR bias, under a consistent protocol, acts as precisely such a constant shift in log-ratio space. This builds a stronger bridge between the intuition and the math.

Thank you for this suggestion. In response, we have added the following intuitive, plain-language explanation of the perturbation-invariant property in the main text.

> Informally, an estimand is perturbation invariant if its value does not change when all samples are subjected to the same compositional perturbation–that is, when their log-ratio representations are shifted by the same vector in log-ratio space.

The reference list is generally good but has a few minor formatting inconsistencies (e.g., journal names in italics vs. not, use of ”et al.”).

Thank you for pointing this out. We have carefully revised the reference list to resolve the minor formatting inconsistencies, such as italicization of journal titles and consistent treatment of ‘et al.’.

References

Justin D Silverman, Rachael J Bloom, Sharon Jiang, Heather K Durand, Eric Dallow, Sayan Mukherjee, and Lawrence A David. Measuring and mitigating PCR bias in microbiota datasets. *PLoS Computational Biology*, 17(7):e1009113, 2021. doi: 10.1371/journal.pcbi.1009113. URL `https://journals.plos.org/ploscompbiol/article?id=10.1371/journal.pcbi.1009113`.

Justin D Silverman, Kimberly Roche, Zachary C Holmes, Lawrence A David, and Sayan Mukherjee. Bayesian multinomial logistic normal models through marginally latent matrix-t processes. *Journal of Machine Learning Research*, 23(7):1–42, 2022.

Emily Van Syoc, Evelyn Weaver, Connie J Rogers, Justin D Silverman, Ramesh Ramachandran, and Erika Ganda. Metformin modulates the gut microbiome in broiler breeder hens. *Frontiers in Physiology*, 13:1000144, 2022. doi: 10.3389/fphys.2022.1000144.
