# Supplementary material for: PCR bias impacts microbiome ecological analyses
Source: PLoS Comput Biol. 2026 Jan 27;22(1):e1013908. doi: 10.1371/journal.pcbi.1013908 (PMC12885373; doi:10.1371/journal.pcbi.1013908)
Supplement: Attachment — Submitted filename: 2025_09_23_Rathod_and_Silverman.docx [file pcbi.1013908.s008.docx]

Summary

Measuring alpha-diversity (within-sample) and beta-diversity (between-samples) are key aspects of analyzing the structure of microbial communities. 16S sequencing is a ubiquitous assay for measuring the composition of these microbial communities, but relies on PCR amplification, which can show differential amplification based on the primers used, resulting in PCR bias. In particular, Non-Primer Mismatch (NPM) persists through all cycles of PCR (while primer-mismatch bias is present only in the initial cycles). Here, Rathod and Silverman use their previously developed model (Silverman et al 2021) to investigate the impact of NPM bias on downstream diversity-based analyses that are frequently used. They prove some fundamental results about which types of measures are susceptible to this bias and demonstrate the possible implications of using these measures.

Overall impression:

The paper is generally well-structured and addresses a fundamental question about the tools used to interrogate the composition of microbial communities. I found this paper to be very compelling, but there are opportunities for improving the presentation of the statistical model and the results.

Major issues:

1. My biggest complaint is the lack of clarity around the simplex figures. What are the defining parameters for the two different scenarios? In figure 2 is it just the orientation of the PCR bias represented by the orange arrows? What is the orange dot? What does it mean for green arrow to “mark the unbiased origin”? Each metric shows bias around the green dot. Does the green dot not represent where each of the taxa are present at a 1/3 proportion? It would be helpful to be explicit around what the PCR bias parameters are in these simulations and what is different between scenarios.
2. I find the data used in this paper to be quite useful and didactic. I am not knowledgeable about current work being done in metagenomics, but is there a scenario where incorrect conclusions have been drawn due to the presence of high PCR bias? Finding such an example and demonstrating that a perturbation invariant measure (or otherwise correcting the analysis) would draw the correct conclusions could be quite impactful. This is a question that could potentially improve the manuscript and not a requirement for publication.

Minor issues:

I have a large number of minor issues, but the majority of them are related to writing/figures and can be resolved with little-to-no additional analyses.

1. The linear log-contrast model notation is a little confusing as written in section 2.1 leading up to equation (2). Rather than introducing everything as vectors and forcing the reader to think about row vs column vectors and then and saying the contrast vector can be viewed as a matrix, simply give the 2x1 matrix formulation from the beginning. Given the multivariate linear model \alpha = x_n \beta and \Sigma, the multinomial GLM is a natural model.
2. In general, I would err on the side of defining every symbol used (\phi for example, is undefined) and making it clear what the dimensions of every matrix are (for example, when taking the vector outer product to obtain an N x D-1 matrix) rather than letting some dimensions be implicitly denoted.
3. You state that you use the fido R library to estimate both \eta and \Sigma. Can you give a short, didactic description of how fido works?
4. For the general audience of PLoS Computational Biology, would be nice to have an intuitive explanation in the text explaining Corollary 1/the perturbation invariant property. Additionally, the theorems/corollaries are quite natural, but their presentation in the theorem-proof manner might turn away the general reader. It might be helpful to intuitively present these results in plain language.
5. Figure 1 does not have quantitative labels for the bias posterior distributions. These would be helpful, at least for the x-axes.
6. Could it be useful to define a relative bias, i.e. the current Bias divided by the estimated log-ratio composition before amplification (and the same question for the beta diversity bias)?
7. Lines 175-180 point to Figure 1, but do not discuss the actual data presented.
8. Lines 181-189 about differential alpha diversity analyses are very interesting in terms of showing how big of a deviation practitioners might experience. It is not necessary to build on this, but I am interested in whether these most extreme differences are typical of what might be observed in practice with real groupings rather than those artificially constructed to be as large as possible.
9. Figure 2 and Figure S2 are very helpful didactic figures (maybe in giving an intuitive explanation of what the perturbation invariance is, you could characterize it in terms of this 3-simplex?), but it is not clear what the two different scenarios consist of in terms of PCR bias (i.e. what is the difference in simulation parameters between left and right?). Perhaps I missed the explanation.
10. As a final comment on the alpha diversity analysis: since all of the alpha diversity metrics presented are perturbation sensitive, does there exist an alpha diversity metric that satisfies realistic desiderata and is perturbation invariant? Not necessary for publication, but this is a natural question. Further, can any of the commonly used perturbation sensitive metrics be “corrected”?
11. Similar to my comments around Figure 1, lines 214-218 only minimally discuss the data presented in Figure 3.
12. I think the beta diversity metrics could also have a relative bias metric to inform the interpretation of how large these biases are.
13. In the discussion, the main guidance for practitioners is to use the Aitchison distance and to be wary when using other metrics. Are there other takeaways? Should the field develop new, invariant metrics? Should we move towards sampling with multiple values of the number of cycles of PCR? Can we correct this?
14. Since PCR is so ubiquitously used, are there other application areas where this work can be extended outside of 16S sequencing?

Other remarks:

1. The GitHub repo should have a license and the readme should have some instructions for running the R scripts
2. Since the log-linear model accounts for “95% of the variation due to PCR bias in both mock and real 16S rRNA libraries”, what accounts for the remaining 5%? Is this 5% safely ignorable, or is it impactful?
3. You state on the main text that Holdemania is underrepresented by a factor of about 32 in Fig. S1, but in Fig. S1, it’s log-ratio is approximately -4 and 2^4 = 16 ,so there is approximately 16x at cycle 35 as at cycle 0. Am I making a simple/obvious mistake in interpreting S1 or should 32 be replaced with 16? For Bacteroides, you state a factor of 4 in the main text and the log ratio is ~2, and 2^2 = 4, so these values agree.
4. I noticed that you implemented the alpha diversity metrics yourself. Is there no R package that implements these?
5. This information is in the caption, but Figure 3 panels A and B could use titles to show which metrics they correspond to.

References:

1. Silverman JD, Bloom RJ, Jiang S, Durand HK, Dallow E, et al. (2021) Measuring and mitigating PCR bias in microbiota datasets. PLOS Computational Biology 17(7): e1009113. <https://doi.org/10.1371/journal.pcbi.1009113>
